# Subset- and Antigen-Specific Effects of Treg on CD8+ T Cell Responses in Chronic HIV Infection

**DOI:** 10.1371/journal.ppat.1005995

**Published:** 2016-11-09

**Authors:** Maria Nikolova, Aurélie Wiedemann, Maria Muhtarova, Daniela Achkova, Christine Lacabaratz, Yves Lévy

**Affiliations:** 1 INSERM, U955, Créteil, France; 2 Immunology Department, National Center of Infectious and Parasitic Diseases, Sofia, Bulgaria; 3 Université Paris Est Créteil, Faculté de Médecine, Créteil, France; 4 Vaccine Research Institute, Créteil, France; 5 AP-HP, Groupe Henri-Mondor Albert-Chenevier, Immunologie clinique, Créteil, France; Emory University, UNITED STATES

## Abstract

We, and others, have reported that in the HIV-negative settings, regulatory CD4+CD25^high^FoxP3+ T cells (Treg) exert differential effects on CD8 subsets, and maintain the memory / effector CD8+ T cells balance, at least in part through the PD-1/PD-L1 pathway. Here we investigated Treg–mediated effects on CD8 responses in chronic HIV infection. As compared to Treg from HIV negative controls (Treg/HIV-), we show that Treg from HIV infected patients (Treg/HIV+) did not significantly inhibit polyclonal autologous CD8+ T cell function indicating either a defect in the suppressive capacity of Treg/HIV+ or a lack of sensitivity of effector T cells in HIV infection. Results showed that Treg/HIV+ inhibited significantly the IFN-γ expression of autologous CD8+ T cells stimulated with recall CMV/EBV/Flu (CEF) antigens, but did not inhibit HIV-Gag–specific CD8+ T cells. In cross-over cultures, we show that Treg/HIV- inhibited significantly the differentiation of either CEF- or Gag-specific CD8+ T cells from HIV infected patients. The expression of PD-1 and PD-L1 was higher on Gag-specific CD8+ T cells as compared to CEF-specific CD8+ T cells, and the expression of these markers did not change significantly after Treg depletion or co-culture with Treg/HIV-, unlike on CEF-specific CD8+ T cells. In summary, we show a defect of Treg/HIV+ in modulating both the differentiation and the expression of PD-1/PD-L1 molecules on HIV-specific CD8 T cells. Our results strongly suggest that this particular defect of Treg might contribute to the exhaustion of HIV-specific T cell responses.

## Author Summary

Protective anti-viral response requires balanced differentiation of effector and memory CD8 T cells. Regulatory T cells with suppressive activity (Treg) provide a protective immune response by differential effects on CD8 T cell subsets. In healthy donors, Treg inhibit the differentiation and proliferation of effectors while preventing the apoptosis of memory cells. These effects are achieved in part by regulating the levels of PD-1/PD-L1 apoptosis-inducing molecules on activated T cells. Despite an expansion of Treg, HIV-1 infection is characterized by a chronic immune activation and”exhausted” HIV-specific effectors expressing high levels of PD-1 and PD-L1. This raised the question about the role of Treg in the HIV+ settings, and the possible therapeutic effect of their depletion. According to our data, the suppressive effect of Treg is highly dependent on the stage of CD8 T cell differentiation, and therefore, is subset- and antigen-specific. Thus, Treg from HIV+ donors inhibit common antigen-specific CD8 effectors, while “exhausted” HIV-specific clones appear resistant to Treg effects. In addition, we show that the subset composition and inhibitory potential of Treg may evolve in the course of infection. These data suggest that the ultimate failure to contain HIV infection results from both an intrinsic impairment of Treg function and a decreased responsiveness of effector clones, associated with high levels of PD-1/PD-L1 expression. Our data also indicate that a simple modulation of PD1/PD-L1 expression on CD8 T effector clones may not restore the protective T cell response.

## Introduction

CD8 T cells play a crucial role in the control of viral infection. While several lines of evidence indicate that HIV-specific CD8 T cells are involved in the response to HIV infection[1,2], their failure to clear the virus in the vast majority of infected individuals has not yet been elucidated[3–5]. Chronic viral infection with ongoing antigenic stimulation may result in exhaustion of virus-specific T cells due to the engagement of down-regulatory mechanisms. Negative pathways involving the programmed cell death-1 (PD-1) and other inhibitory receptors such as CTLA-4, Tim-3 and LAG-3 can be critical in limiting the magnitude or duration of the antiviral CD8 T-cell response[6–8]. In human, the phenotype and functional potential of chronically stimulated CD8 T cells differ among viral infections (9).HIV-specific response is characterized by a decreased proliferative capacity of the central memory CD8 T cell pool, incomplete differentiation of effectors with reduced cytotoxic ability, and increased sensitivity to apoptosis as compared to CD8 T cells specific for non-progressive persistent infections, such as EBV or cytomegalovirus (CMV)[10,11].Several studies have suggested a particular role for the PD-1/PD-L1 pathway in the exhaustion of HIV-specific CD8 T cells. PD-1 is upregulated on HIV-specific [12] and simian immunodeficiency virus (SIV)-specific CD8 T cells [13], and the level of PD-1 expression is associated with decreased HIV-specific CD8 T-cell proliferation [14]. The frequency of PD-1 expression on HIV-specific CD8+ T cells was shown to parallel the levels of plasma HIV viral load, while negatively correlating with CD4+ T cell counts [12]. Furthermore, in non-human primate model, in vitro blockade of PD-1 on CD8 T cells enhanced the proliferation and cytokine production of antiviral CD8 T cells, suggesting that immune exhaustion was reversible[15]. Finally, a recovery of antigen-specific CD8 T cells from exhaustion has been demonstrated in vivo after blocking the PD-1/PD-L1 interaction in a murine model of LCMV infection, as well as in a SIV-macaque model. [13,16].

In parallel to the “exhausted” CD8 T cell response, an expansion of regulatory CD4+FoxP3+ T cells with suppressive activity (Treg) is well established in the chronic phase of HIV infection as well as in other chronic infections [17–23], Treg have a key role in maintaining peripheral tolerance, and limiting chronic inflammatory responses. In the healthy immune system, we and others have shown that Treg are responsible for balanced differentiation of memory and effector CD8 T cells in case of strong stimulation, thus providing a protective immune response [24,25]. We have shown that Treg may regulate differentially the apoptosis of memory and effector CD8 T cells through the PD-1/PD-L1 pathway, by regulating the levels of PD-L1 expression [25].Gal-9,another inhibitory receptor constitutively expressed by Treg may provide the ligand for selective inducing of tolerance in Tim-3–expressing effectors [26].

In the settings of chronic infection, Treg may be either beneficial through limiting non-specific immune activation, or have detrimental effect by suppressing effective antigen-specific immune responses [27]. It seems that disease outcome depends on the equilibrium between a balanced Treg to effector T-cell response and immune activation [28]. Indeed, the increased level of Treg in HIV+ patients has been linked to a reduced immune activation and low viral load [29,30] but the accumulation of Treg in lymphoid tissues was also found to be associated with a high viremia, both in infected patients and SIV-infected macaques [31,32]. In addition, high Treg levels have been associated with poor immune restoration under ART [33].

Thus, the role of Treg in chronic HIV infection remains an open question. Moreover, the subset-specific effects of Treg on CD8 responses of HIV+ patients and the underlying mechanisms have not been evoked yet. Logically, in conditions of prolonged stimulation and effector T cell exhaustion, the mechanisms and molecules implicated in Treg-mediated inhibition would also evolve, or be perturbed. Recent data have challenged the notion of Treg as a homogeneous, and stable population, suggesting that, under inflammatory conditions, they may acquire different phenotype and functions [34–36].As a consequence, the therapeutic potential of Treg depletion, whether combined or not with blocking of PD-1/PD-L1 or other signaling checkpoints to activate a potent anti-viral immune response, is still an unresolved issue of utmost interest.

In this paper, we studied the inhibitory effects of Treg on CD45RA/CCR7-defined naïve, memory and effector CD8 T subsets in HIV+ subjects after polyclonal and antigen-specific stimulation. Our results provide evidence that, in conditions of chronic HIV infection, Treg may exert subset- and clonally- specific inhibitory effects on CD8 T cells. These results indicate that the ultimate failure to contain generalized HIV-1–associated immune activation is a joint result of intrinsic impairment of Treg function and decreased responsiveness of the effector clones, at least in part associated with the high levels of PD-1/PD-L1 expression.

## Results

### Treg inhibit the proliferation and differentiation of effector CD8 T cells in chronic HIV infection

**Fig 1:**
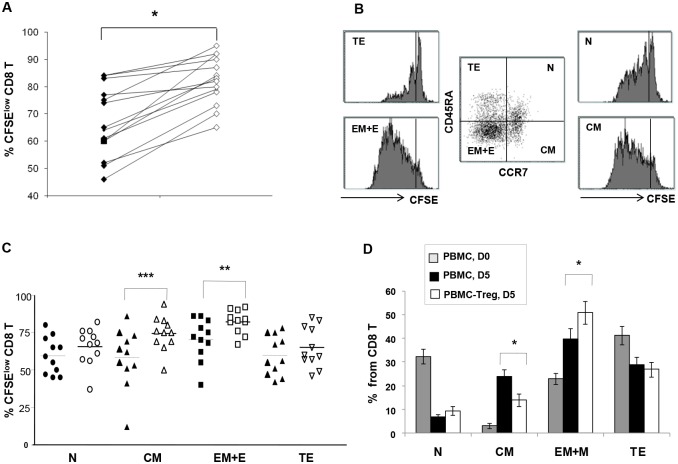
Treg inhibit the proliferation of CD8 T cells from HIV+ patients in a subset-specific manner. **A.** Individual data (n = 14) of proliferation rates of CD8 T cells after polyclonal stimulation (5d, anti-CD3) in the presence (black) or in the absence (white)of Treg. **B.** Representative dot-plot of CD8 T cell-gated PBMC showing the CCR7/CD45RA defined subsets: naïve (N), central memory (CM), effector-memory and effector (EM+E), terminal effector (TE), at D5 of anti-CD3 stimulation, and their further analysis according to CFSE intensity. **C**. Individual data ofproliferation rates of CCR7/CD45RA-defined CD8 T (CD8 N, CM, EM+E, and TE) subsets (**P<0.01, ***P<0.001, n = 11, Student’s T-test). **D**. Proportions of CCR7/CD45RA-defined CD8 T subsets before (grey) and after polyclonal stimulation, in the presence (black) or in the absence (white) of Treg. Bars correspond to mean ±SD, n = 11, * P<0.05, Student’s T-test.

First of all, using PBMC from n = 14HIV-1-positive subjects under ARTwe confirmed the inhibitory effect of Treg on the proliferation of global CD8 T cells after polyclonal stimulation (coated anti-CD3 for 5 days). The percentage (mean ± SD) of proliferating (CFSE^low^) CD8 T cells on D5 was 65 ± 12% in the presence of Treg (total PBMC) vs. 82 ± 8.9% after Treg depletion, P<0.01 (Fig 1A).Next, we compared the proliferation of CCR7/CD45RA-defined CD8 T subsets: naive (N, CD45RA+CCR7+), central memory (CM, CD45RA- CCR7+), effector memory+effector (EM+E, CD45RA- CCR7-) and terminal effector (TE, CD45RA+ CCR7-) (Fig 1B). The CD8 TCM subset contained (mean ± SD) 57 ± 20% CFSE^low^ cells in the presence of Treg vs. 74 ± 12% in their absence (P<0.01), and the CD8 EM+ E subset, 69 ± 14% vs. 79± 6%, respectively, P<0.001. At the same time, the share of CFSE^low^ cells within the N and TE pools was not affected significantly by the presence or absence of Treg (Fig 1B and 1C).These data showed that the inhibitory effect of Treg on CD8 T cells was subset-specific.

Since polyclonal proliferation may be accompanied by differentiation, CFSE^low^ CD8 T cells detected on D5 may have originated from N, M or E subsets. To clarify this issue we compared the proportions of CD8 subsets before and after stimulation, in the presence or in the absence of Treg. Polyclonal stimulation increased the proportions (mean%) of CM and EM+E subsets at the expense of N and TE CD8 T subsets. More detailed analysis showed that the increase of CM subset was more important in the presence of Treg (from 4% to 22%) as compared to their absence (14%) (P<0.05). The increase of the EM+E subset was less significant in the presence of Treg (from 22% to 40%) than in their absence (51%) (P<0.05 (Fig 1D).These results suggest that in chronic HIV infection Treg exert differential effects on the CD8+ T cell subsets and prevent the differentiation of CM cells towards the effector-memory stage.

### Treg-mediated inhibition of CD8+ T cell responses in ART- HIV+ patients differs at the clonal level

**Fig 2:**
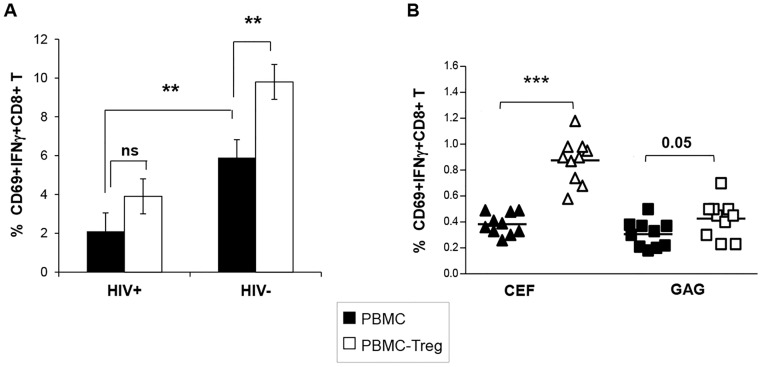
CD8 T cell sensitivity to Treg-mediated inhibition differs at the clonal level. **A.** Pooled data showing the effect of Treg on the IFN-γ secretion of polyclonally stimulated CD8 T cells in 10HIV-1+ and in 10HIV- subjects. Bars represent mean ± SD. **B.** Individual data showing the percentage of IFN-γ secreting CD8 T cells after peptide-specific (CEF or Gag) stimulation of total and Treg-depleted PBMC from HIV+ ART- patients. (* P <0.05, ** P<0.01, *** P<0.001, n = 10, paired Student’s T-test).

Next, we studied Treg-mediated effects on the production of IFN-γ by CD8 T cells after polyclonal stimulation of PBMC from ART-naïve HIV+ patients, or HIV- controls. As expected, overnight anti-CD3 stimulation of total PBMC from HIV+ subjects resulted in lower differentiation of IFNγ+ producing cells in PBMC form HIV+ subjects as compared to PBMC from HIV- controls (mean ± SD % of CD8+CD69+IFNγ+ cells of 2.1 ± 0.95 vs. 5.9 ± 0.92, P <0.05; n = 10). Treg depletion increased the percentage of IFNγ-secreting CD8 T cells in HIV-negative controls (mean 9.8 ± 0.9%), (P<0.01 for comparison with cultures with Treg) but not in HIV+ patients (mean 3.9 ± 0.9%), (P = NS) (Fig 2A).In the settings of non-ART-treated patients, IFNγ expression in total PBMC was mostly confined to the EM+E subset (S1B Fig, embeded), while in healthy controls, IFNγ was mostly expressed by CM CD8 T cells (S1A Fig, embeded). Treg inhibitory effect was observed for all subsets in HIV-negative settings, reaching significance for CM an EM+E subsets, while in HIV+ donors it was observed only at the level of CM IFNγ-producing cells, confirming our hypothesis about differential effects of Treg in HIV settings (S1 Fig). In fact, these results might suggest that in conditions of HIV infection, CD8 T cells were defective, not only in terms of IFNγ production but also in sensitivity to Treg-mediated suppression, and/or Treg could be less suppressive.

It is well known that memory CD8 T cells differ in phenotype depending on their specificity. Therefore, we further compared the inhibitory effect of Treg from ART naive-HIV+ patients on autologous CD8 T cells having different antigen specificity. To this end, PBMC were stimulated overnight with HIV Gag or CEF peptides, either in the presence or in the absence of Treg. Treg depletion had a non-significant effect on the frequency of HIV Gag-specificCD8+IFNγ+T (mean% 0.3 vs. 0.45 with or without Treg, respectively; P = 0.05) while the frequency of CEF-specific CD8+IFNγ+ T from the same patients increased significantly (0.38 vs. 0.87%, P < 0.001), (Fig 2B). We concluded that Treg inhibitory effects observed in HIV infection differed depending on the CD8 T cell specificity.

### Treg from HIV infected patients do not modulate the expression of pro-apoptotic molecules on autologous CD8 T cells

**Fig 3:**
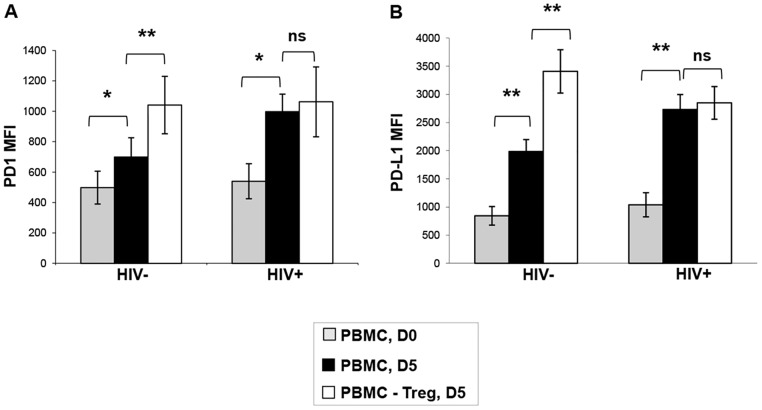
Treg from HIV infected patients do not modulate the expression of pro-apoptotic molecules on autologous CD8 T cells. Pooled data about the expression levels of PD-1 (**A**) and PD-L1 (**B**) on CD8 T cells from HIV+ or HIV- subjects, before (grey) and after 5 days of polyclonal stimulation in the presence (black) or in the absence (white) of Treg. (n = 6, ** P<0.01, Student’s T-test).

Activated CD8 T cells express increasing levels of pro-apoptotic molecules, such as PD-1 and its ligand, PD-L1. We have previously shown that, in healthy donors, Treg-mediated inhibition of PD-L1 expression rescued preferentially the PD-1^high^ CM CD8 subset and impacted the balance M/E cells [25]. To check whether this regulatory mechanism operated in HIV infection, we compared the expression of PD-1 and PD-L1 on total CD8 T from HIV+ART- patients, and from HIV-controls, before and after 5 days of polyclonal stimulation, in the presence or in the absence of Treg. The baseline expression levels (mean MFI) of PD-1 and PD-L1 on the global CD8 T population from HIV-negative and HIV+ donors were not significantly different 498±108 vs. 540±115 for PD-1 and 844±176 vs. 1040±202 for PD-L1, respectively (Fig 3A and 3B). After polyclonal stimulation, the expression levels of PD-1 on CD8 T cells rose significantly to 699±127 and 998±115 in HIV- and HIV+ donors, respectively (P<0.05 for both comparisons to unstimulated conditions). Similarly, these increases were 1985±186 and 2730±305for PD-L1 expression (P<0.01 for both comparisons to unstimulated conditions). In the HIV-negative settings, stimulation in the absence of Treg resulted in significantly higher levels of PD-1 and PD-L1 on the activated CD8 T cells, as compared to stimulation in the presence of Treg: 1041±189 vs. 699±127 for PD-1 and 3407±116 vs. 1985±176 for PD-L1 (P<0.01 for both comparisons). Interestingly, Treg depletion had no such effect in HIV+ donors: 1062±230 vs. 998±115 for PD-1, and 2848±320 vs. 2730±305 for PD-L1 respectively, (P>0.05 for both comparisons), (Fig 3A and 3B). In addition, we studied the levels of Annexin V+ cells within N, CM, EM+E and TE CD8 subsets from HIV+ donors (n = 6) after 48h anti-CD3 stimulation, either in the presence of Treg or after their depletion. No significant difference was detected, indicating that CD8+ T cells from HIV-infected patients are not influenced by the suppressive effect of Treg in terms of expression of PD-1/PDL-1 and activation-induced apoptosis (S2 Fig).

**Fig 4:**
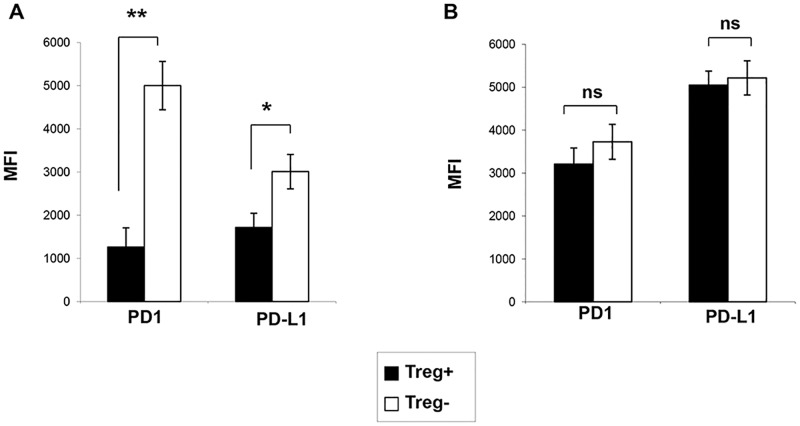
Treg-mediated regulation of PD-1/PD-L1 expression on CD8 T cells from HIV+ patients is clonally-specific. Pooled data on the levels of PD-1 and PD-L1 expression on CEF-specific (A) and Gag-specific (B) CD8 T cell effectors stimulated overnight in the presence (black) or in the absence (white) of Treg. (* P <0.05,*** P <0.001, n = 10, paired Student t-test).

Next, we investigated whether Treg effects on the expression of PD-1 and PD-L1 were dependant on the antigen specificity of effector cells. To this end, the expression of PD-1 and PD-L1 on the IFNγ+ CD69+CD8 T cells of HIV+ ART- donors was studied after 18h stimulation with CEF or HIV Gag peptides, as described in M&M section. CEF-specific CD8 T effectors expressed moderate levels of PD-1 and PD-L1, that increased significantly after Treg depletion (MFI 1263 vs. 5003, P<0.01 for PD-1, and 1720 vs. 3060 for PD-L1, P<0.05 for both comparisons), (Fig 4A). A similar effect was noted on CEF-specific CD4 T cells following Treg depletion with an increase from 891 to 1918 for PD-1MFI and 1449 to 2862 for PD-L1 MFI,(P<0.05 for both comparisons, S3A Fig).

As expected, HIV Gag-specific CD8 T cells expressed significantly more pro-apoptotic molecules as compared to the CEF specific CD8 T cell clones at baseline: 3208 vs. 1263 for PD-1 and 5049 vs. 1720 for PDL1 (P<0.05 for both comparisons) (Fig 4B). Noteworthy, this expression did not change significantly after Treg depletion: 3727 and 5216 respectively for PD-1and PDL1 (Fig 4B). Similarly, HIV Gag-specific CD4 T cells expressed comparable levels of PD-1 and PD-L1, regardless of the presence or absence of Treg: 2920 vs. 3037, and 4734 vs. 4851, respectively (S3B Fig). Altogether, these data showed that the functional inhibitory effects of Treg on T cells, as well as their regulatory effects on the expression of PD-1 and PD-L1 differ at the specificity level.

### Treg/HIV+ lack suppressive capacity as compared to Treg/HIV- and fail to modulate PD-1/PD-L1 expression on HIV-specific CD8 T cells

**Fig 5:**
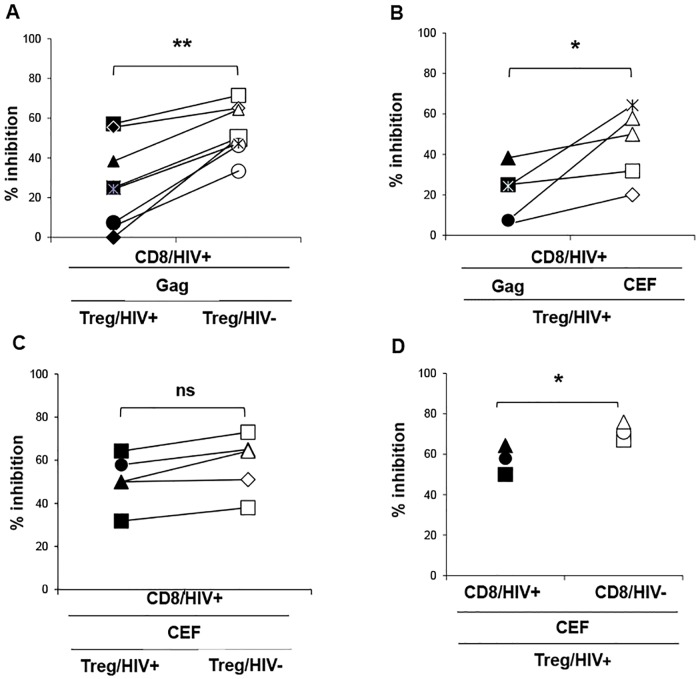
Treg/HIV+ lack suppressive capacity as compared to Treg/HIV- and fail to modulate PD-1/PD-L1 expression on HIV-specific CD8 T cells. Individual data from co-culture and cross-culture studies comparing the inhibition of IFNγ expression by **A.**Gag-stimulated HIV+ CD8 T cells in the presence of autologous (left) or HIV- (right) CD4+CD25^high^ T cells, (n = 8).**B.** Gag-stimulated (left) and CEF-stimulated (right) HIV+ CD8 T cells in the presence of autologous CD4+CD25^high^ T cells, (n = 5). **C**. CEF-stimulated HIV+ CD8 T cells in the presence of autologous (left) or HIV- (right) CD4+CD25^high^ T cells, (n = 5).**D.** CEF-stimulated autologous co-cultures and cross-cultures in which HIV+ CD8 T have been replaced with CD8 T from HIV- donors, (n = 3; * p<0.05, Student’s T-test).

To explain the observed antigen-specific effects, we wondered whether Treg from HIV+ patients (Treg/HIV+) were functionally deficient, therefore failing to regulate the excessive differentiation, and the expression of proapoptotic molecules by HIV-specific clones. To this end, we set co-cultures with purified CD8 T cells and Treg from HIV+ART- patients, in the presence or in the absence of HIV Gag, or CEF antigens. In cross-over cultures, either the CD8 T cells or Treg were replaced with those from HIV- controls (Treg/HIV-). IFNγ expression by CD8 effectors as well as PD-1 and PD-L1 expression levels on the effector and on the regulatory T cells were measured 18h later. According to our results, Treg/HIV- suppressed the expression of IFNγ by Gag-specific CD8 T better than patients’ Treg (mean IFN γ inhibition% of 53±12 vs. 27±22; P<0.05). Although the suppressive effect of Treg/HIV+ varied considerably between patients, replacement with Treg/HIV- invariably resulted in a more important inhibition, indicating a decreased inhibitory potential of Treg in the settings of chronic HIV infection (Fig 5A). In a series of experiments we demonstrated the direct effect of HIV viral load on Treg function. Treg from patients receiving ART, and with confirmed viral suppression, had a significantly increased inhibitory potential on Gag-specific CD8 T response as compared to Treg isolated from patients with active viral replication. This was observed either in autologous conditions (co-cultures of CD8 T cells from untreated patients with Treg from same patients after ART treatment) or in allogenic conditions (Treg from other patients under ART) (mean IFNγ inhibition % of 34 vs. 15, respectively, MW p<0.01),(S5 Fig).

Next we investigated whether this defect of Treg/HIV+ concerned also non HIV-specific T cells.

Results showed that Treg/HIV+ inhibited the autologous CEF-specific CD8 T much better than Gag-specific ones (average inhibition %, [min–max]: 45, [18–64]) vs. 21[7–38], P< 0.05(Fig 5B) and similarly to Treg/HIV- in cross-over cultures (51 [32–64] vs. 58, [38–73], P> 0.05), (Fig 5C). Further on, the PD-1 MFI on the CEF-specific CD8 T cells decreased significantly from 3504 to 2465 and 2595 in the presence of Treg/HIV+ and Treg/HIV-, respectively (P<0.05 for both comparisons, (S4A Fig). Similarly, PD-L1 MFI decreased from 8900 to 6155 and 5748, respectively, (P< 0.01 for both comparisons,(S4B Fig). At the same time, no significant difference was noted between the expression levels of PD-1 and PD-L1 on Gag-specific effectors, whether in the presence of Treg /HIV+ or Treg/HIV- (MFI for PD-1: 4642 without Treg vs. 3535 and 3595, in the presence of Treg/HIV+ and Treg/HIV-, respectively; MFI for PD-L1: 11047 without Treg vs. 9425 and 8931, in the presence of Treg/HIV+ and Treg/HIV-, respectively (P>0.05 for all comparisons, S4 Fig). Finally, Treg isolated from three HIV+patients suppressed CEF-specific CD8 T-cells from HIV- controls much better than CD8 T-cells from HIV+ patients (mean inhibition % of IFNγ expression 71±5 vs. 57±7 respectively; P<0.05),(Fig 5D).

In summary, these results showed a defect in the regulation of CD8 T cell proliferation, differentiation and function in HIV infected individuals. We show that the suppressive capacity of Treg is impaired in HIV-infected patients as compared to those from non HIV-infected individuals. Moreover, this impairment is dependent on the specificity of CD8 T cells. The lack of Treg-mediated effects on the expression of PD-1/PD-L1 molecules on HIV-specific CD8 T cells, in contrast to other recall antigen-specific clones from the same patients, strongly suggest that this particular defect of Treg might contribute to the exhaustion of HIV-specific T cell responses.

## Discussion

In this study we explored the suppressive effects of Treg on CD8 T cell responses in chronic HIV infection. The present data extend significantly our previous results in non-infectious context [25] showing that the suppressive effect of Treg is highly dependent on the stage of CD8 T cell differentiation. Likely, these data contribute to explain that the ultimate failure to contain generalized HIV-1–associated immune activation in HIV+ infection results from both an intrinsic impairment of Treg function and a decreased responsiveness of effector clones, at least in part associated with the high levels of PD-1/PD-L1 expression.

First, we demonstrate that Treg from HIV+ individuals display lower suppressive effects as compared to those from HIV- controls. Second, regarding the effects of Treg on particular CD8 T cell populations, we show clearly that Treg suppress mostly the differentiation of effector-memory and effector CD8+ T cells in HIV-infected patients. Third, HIV-specific CD8+ T cells were less sensitive to the suppressive effects of Treg as compared to other recall antigen-specific CD8+ T cells. Finally, HIV-specific effector CD8 T cells are resistant to Treg—mediated modulation of the “exhaustion”molecules PD-1/PDL-1.

Numerous studies have shown that Treg expand during acute and chronic HIV-1 infection, and inhibit effector CD8 T cell responses in vitro [17,21,37]. Recent data about the heterogeneous nature of Treg and their plasticity indicate that Treg effects may differ depending on the phase of immune response, the nature of stimulus and the differentiation state of the target subsets.

In the HIV-negative settings, Treg exert differential effects on the proliferation, maturation and apoptosis of CD8 subsets and in this way maintain the memory/effector cell balance in case of strong stimulation [25]. Globally, we show here that Treg from HIV+ donors with controlled viral load inhibited the differentiation of naïve CD8 T cells into CCR7- CD45RA- EM+E cells, and favored the accumulation of cells with CM (CD45RA-+CCR7+) phenotype. The memory-like phenotype of HIV-specific CD8 cells combined with decreased proliferative and cytokine-secreting capacity, has been well documented [3,5,11].We show for the first time that these characteristics of the HIV-specific pool may result from the subset-specific homeostatic effects of Treg that have become irrelevant in conditions of generalized and lasting immune activation.

In addition to the subset-specific effects of Treg, we demonstrate that Treg-mediated inhibition differs depending on the antigen specificity of CD8 response. In our hands, the effect of HIV+ Treg on HIV Gag-specific clones was significantly inferior to the inhibitory effect on CEF-specific cells, whether from HIV+ or HIV- donors. One logical explanation is the differing sensitivity of the responding cells to Treg-mediated inhibition. In healthy donors, CD8 cells specific for common viral infections as CMV, EBV, and flu are composed mostly of non-activated memory cells [9,38]. In the HIV+ settings, virus-specific cells are either in a state of immune senescence as a result of increased susceptibility to co-infections [39,40] combined with frequent by-stander activation [41], or—exhaustion resulting from continuous stimulation in the settings of high antigen load, and characterized by progressive loss of effector functions[42]. Therefore“exhausted” HIV-specific CD8 would be less capable to produce IFNγ as compared to the “senescent|” non-HIV specific ones, and Treg depletion may not restore their functionality.

Increased expression of the co-inhibitory programed cell death-1 (PD-1) receptor is a hallmark of exhausted T cells [12,14,43]. Studies in animal models and in humans have pin-pointed PD-1/PD-L1 pathway as crucial for the outcome of a virus-specific response, independently of CD4+ T cell help [14,44–46].The levels ofPD-1 and its ligand correlate with the antigen burden and the duration of stimulation as shown in HIV, HCV, and HBV chronically infected patients [14,18,45,46]. Activated CD8 T cells engaged towards CM differentiation express the highest levels of PD-1 and are therefore much prone to “exhaustion”[14]. In the HIV-negative settings, CM CD8 could be rescued from activation-induced apoptosis through Treg-mediated regulation of PD-L1 expression, both on by-stander CD8 and on Treg themselves [25]. Here, we show that in HIV-infected donors this regulatory mechanism is operating only with respect to non-HIV specific clones. No such effect was observed with Gag-specific CD8, implying that Treg are losing their ability to regulate CD8 response through PD-1/PD-L1 pathway in case of immune exhaustion.Further, ART-mediated viral suppression may not readily revert high PD1 and PD-L1 expression on CD8 T cells. In our hands, PD1 and PD-L1 expression was not significantly diminished when replacing autologous Treg with Treg from settings with undetectable VL (S5 Fig). In line with our observations, it was shown that the reversible regulation of PD-1 expression may be eventually lost, as in HIV infected subjects, PD-1 levels on CD4 and CD8T cells remain high following anti-retroviral therapy[43].

In addition to a differential sensitivity of the regulated clones, we have demonstrated that the suppressive potential of Treg changes in the HIV+ settings. Treg are directly affected by HIV, and may experience variable extent of impairment. In different models of chronic stimulation, Treg survival and functions closely depended on PD-1 [47,48], as well as on PD-L1 expression [47,49]. In our hands, autologous Treg inhibited HIV-specific responses less well than did Treg from HIV-negative donors. The correlation between the viral load and Treg inhibitory effect in the studied group of HIV+ART- patients did not reach significant values. However, ART-mediated suppression of HIV VL significantly restored Treg function, which might be in part explained by a significantly decreased expression of apoptosis-inducing receptors on Treg themselves (S6 Fig).

Data on the quality and evolution of Treg-mediated inhibition in chronic infection are limited and contradictory. Studies on peripheral Treg have found a decreased suppressive capacity of Treg from patients with progressive HIV infection [21,37], while two recent studies reported preserved suppressive capacity of peripheral blood and mucosal Treg in non-controlled HIV-1 infection [31,50]. However, both studies have assessed Treg inhibitory effect on the proliferation of anti-CD3 stimulated non-Treg CD4+ T cells, and not on antigen-specific CD8 T cell responses. Evenmore so, Shaw et al. [31] observed a trend toward increased suppression by mucosal Treg from noncontrollers regarding one particular effect: OX40 co-expression by non-Treg CD4 T.This was associated with significantly increased CD38 expression by mucosal Treg. Globaly, these results may indicate that Treg can modulate effector functions to different degrees dependingon the tissue microenvironment and specific pathways engaged. The genuine heterogeneity of Treg has just started to be elucidated. Three phenotypically and functionally distinct Treg subpopulations have been defined: CD45RA+FoxP3^low^ naive, CD45RA-FoxP3^high^effector, and a CD45RA-FoxP3^low^ non suppressive subset [51]. Arecent study showed that it was the effector Treg that were consistently affected during HIV infection, and were negatively correlated with the magnitude of HIV-specific CD8 T-cell responses [52]. According to our depletion protocol [27], we have characterized mostly the function of the effector Treg subset. The observed higher inhibitory effect of Treg from HIV-negative donors on Gag-specific cells (S5 Fig) was probably due to a higher effector/naïve Treg subset ratio as compared to HIV+ Treg.In line with this hypothesis, preincubation with Gag peptidesenhanced the suppressive effect of HIV+ Treg by inducing the differentiation of naïve Treg, or of non-Treg CM or EM cells [53,54] or either by inducing the apoptosis of PD-1^high^non-functional Treg. Similar effects of low-dose antigen stimulation on the generation of Ag-specific adaptive Treg have been reported in other *in vitro* models [55,56].

In line with the present results, we have previously reported that in ART- chronic HIV+ patients, the suppressive activity of CD4+CD25+ T on the proliferation response to tuberculin did not vary over time while the suppressive activity in response to p24 decreased significantly in the course of 24 months [37]. Globally, these data consistently demonstrate that the suppressive efficiency of Treg decreases in the settings of chronic infection in a clonally-specific manner.

Therefore, we support the hypothesis that the ultimate failure to contain generalized HIV-1–associated immune activation, even in ART treated HIV+ patients, is a joint result of intrinsic impairment of Treg function and decreased responsiveness of the effector clones, at least in part associated with the high levels of PD-1/PD-L1 expression.

Elimination of Treg in the settings of chronic infection has been considered as an attractive approach to restore virus-specific CD8 responses [57]. We have recently shown that responses to the administration of a therapeutic vaccine in HIV+ infected patients are influenced by the frequency of HIV-specific Treg at baseline, and vaccinees who displayed lower levels of HIV-specific Treg responded better to the therapeutic vaccine [58].Yet, a simple elimination of Treg would also abrogate their homeostatic effects leading to autoimmune phenomena.

Blockade of inhibitory receptors also results in functional recovery of exhausted T cells; the most convincing examples including PD-1 blockade in HIV, HBV, and HCV infection [13,47,59–61]. However, repeated anti-PD-1 treatment may increase the likelihood of autoimmune events just as Treg depletion does. PD-1/PD-L1 blockade might reactivate Treg as well, and compromise the reactivation of antigen-specific response. Even if viral load suppression, followed by PD1/PD-L1 blockade could restore virus-specific response, the containment of low-level immune activation may prevent the complete restoration of Treg proliferation or suppression function [62]. Here, we prompt the possibility to improve the composition and functionality of the Treg pool through selective modulation of Treg subsets. Treg subsets could respond differently to external signals, and therefore—be modulated, using blocking Abs, chemical products or cytokines [34,35,58]. We have recently shown a differential in vitro effect of IL-7 on purified Treg subsets from HIV-negative individuals, and confirmed that IL-7 therapy in HIV+ infected subjects impacted also Treg function [34].

In summary, our present results demonstrate subset- and antigen-specific Treg effects in the settings of chronic HIV infection. These observations point out also a defect in the suppressive capacity of Treg which may explain the persistence of immune activation in HIV+ treated patients. Finally, these data may help the further precision of immune-intervention therapies.

## Materials and Methods

### Patients and cell populations

Blood samples from HIV-1-positive subjects, either naive from treatment (ART-, n = 10, mean CD4+ T cells counts 545 ± 158 /μl; mean viral load 3.9 ± 1.1 log HIV RNA copies /ml), or under ART (n = 14, mean CD4+ T cells counts 675 ± 242 cells; viral load < 1.6 log HIV RNA copies /ml), were collected at the Clinical Immunology Department of Henri Mondor Hospital Créteil, France and the National Center of Infectious and Parasitic Diseases, Sofia, Bulgaria, in the course of routine immune monitoring. Blood samples from HIV-negative healthy donors were obtained at the Regional Blood Transfusion Centre, Creteil, France.The research was conducted in accordance with the Declaration of Helsinki. The study was approved by the respective ethical committees, and written informed consent from all subjects were obtained before study initiation.Peripheral blood mononuclear cells (PBMC) were isolated using standard gradient separation technique. Half of them were Treg-depleted, using anti-CD25 Dynabeads and a Dynall magnetic separation system, after the manufacturer’s protocol (Invitrogen Dynal AS, Oslo, Norway). The Treg-depleted fraction contained < 0.1% CD4 T expressing high levels of CD25 and FoxP3 transcription factor as verified by flow cytometry (S8 Fig). In a limited number of experiments total CD8 T cells, and CD4+CD25^high^ Treg fractions were obtained from frozen or fresh PBMC, using negative isolation kits from Miltenyi Biotec (Bergisch-Gladbach, Germany) according to the manufacturer's instructions, as previously described. The purity of purified populations was >95%. Treg cells were defined as CD4+CD25^high^FoxP3+CD127^low^T cells as we have previously reported [25].

### Proliferation assays

PBMC/Treg+ and PBMC depleted in Treg (Treg- fraction) from HIV-1+ART+ patients were stained with CFSE (Molecular probes, Eugene OR, US) at final concentration 0.5 μM and cultivated in 96-well U-bottom plates, coated with 5 μg/mL anti-CD3 mAb (UCHT1; Beckman Coulter, Villepinte, France); (total cell concentration 1.25 x 10^5^/ml and final volume 200 μl; Treg/effector ratio 1:4 as previously described [25]). On day 5 (D5) cells were washed and surface-stained with a combination of CD27/CD8/CD45RA/CCR7/CD3 mAbs. CD8 T cell subsets were defined as follows: CCR7+CD45RA+ (naïve, N), CCR7+CD45RA- (central-memory, CM), CCR7-CD45RA- (effector-memory + effector, EM+E), and CCR7-CD45RA+ (terminal effector, TE). Proliferation rates were assessed according to the percentage of CFSE^low^ cells at D5 by multicolor flow cytometry (FACSCanto, BD).

### Intracellular cytokine production and apoptosis studies

For intracellular cytokine staining (ICS), overnight stimulation of PBMC (either Treg-depleted or not) was carried out in plates coated with anti-CD3 (5 μg/ml) or with a pool of whole Gag 15-mer peptides (2 μg/ml) supplemented with anti-CD28 and anti-CD49d antibodies (1 μg/ml of each), or control CEF (CMV, EBV and influenza virus) peptides (5μg/ml each). Brefeldin A (10 μg/ml) was added 1h after the peptide stimulation. Cells were surface stained with anti-CD8, anti-CD4, anti-CD69, anti-PD-1 and anti-PD-L1 mAb and ICS was performed with PE-Cy7- or FITC-conjugated IFNγ antibody. For co-culture studies CD8+ T cells (5 × 10^5^ cells/well) were cultivated in 48-well plates, in the presence or absence of autologous or allogeneic CD4+CD25+ T cell fractions at a ratio 4:1, and autologous monocytes (10^4^/well.) In a limited number of experiments, the CD4+CD25+ T cell fraction was stimulated with Gag 15-mer peptides (2 μg/ml) in the presence of autologous monocytes (10^4^/well.) for 18h before the setting of co-cultures.For apoptosis studies, PBMC Treg+ and PBMC Treg- fractions from HIV-1+ART+ patients and from HIV- controls were cultivated in 96-well round-bottom plates coated with anti-CD3 at 5 μg/mL. Forty-eight hours later, cells were harvested, washed with cold PBS, suspended in binding buffer (containing 2.5 mM CaCl_2_), and stained with annexin V–FITC (apoptosis kit; BD Pharmingen) and a combination of CD27-PE/CD8-PerCP/CD45RA-APC mAbs for 20 minutes on ice. At the end of the culture, 300 μL of binding buffer was added, and the samples were analyzed within 30 minutes by flow cytometry while kept on ice.

### Immunofluorescence and flow cytometry

Anti-CD25-APC-Cy5, anti-CD127-Alexa-Fluor700, anti-CD4-FITC or Pacific blue, anti- CD45RA-FITC or PE-Cy5, anti-CCR7-PE, or PE-Cy7, anti-CD8-PerCP, anti-CD3-AmCyan, anti-IFN-gamma-FITC or APC-Cy7, anti-CD69-APC, anti-PD-1-PE and anti-PD-L1-PECy7, anti-FoxP3-PE were all products of BD Biosciences. Cells were analysed by FACSCanto II, and LSR II (BD Immunocytometry systems). At least 20 000 CD4 or CD8-gated events, and at least 100000 CD4 or CD8-gated events were collected for cell surface and intracellular studies, respectively.

### Statistical analysis

Flow cytometry data were analyzed by FACSDiva v.6.1.2. and FlowJo software. Statistically significant differences were assessed by one-way ANOVA, followed by paired T-test, or by unpaired T-test assuming independent samples where appropriate. Correlations were assessed using Spearman’s rank order test (GraphPad° Prism 5.0 statistical software).

## Supporting Information

S1 Fig Effects of Treg on the expression of IFNγ by CD8 T cells at the subset level. Individual data showing the effect of Treg on IFNγ secretion within CCR7/CD45RA-defined CD8 T subsets, after 18-hour stimulation with anti-CD3 antibodies in the presence (black) or in the absence (white) of Treg, in HIV- negative (A) and in HIV+ART-naïve (B) subjects. Embeded: pooled data showing the distribution of IFNγ+ CD8 T cells within the CD45RA/CCR7 defined subsets in HIV-(left) and in HIV+ settings (right). Bars represent mean ± SD. (* P <0.05, ** P<0.01, *** P<0.001, n = 10, paired Student’s T-test).
(TIF)

S2 Fig Activation-induced apoptosis escapes from Treg control in HIV+ settings. Pooled data about the percentage of AnnexinV+ cells within CD27/CD45RA-defined CD8 T subsets, after 48 hour stimulation with anti-CD3 antibodies in the presence (black) or in the absence (white) of Treg.(ns P >0.05, (n = 6, Student’s T-test).
(TIF)

S3 Fig Treg-mediated regulation of PD-1/PD-L1 expression on CD4 T cells from HIV+ patients is clonally-specific. Pooled data on the levels of PD-1 and PD-L1 expression on CEF-specific (A) and Gag-specific (B) CD4 T cells stimulated overnight in the presence (black) or in the absence (white) of Treg. (* P <0.05,*** P <0.001, n = 10, paired Student t-test).
(TIF)

S4 Fig The failure of Treg/HIV+ to modulate PD-1/PD-L1 expression depends on the antigen specificity of CD8 T cells. Individual data from co-culture and cross-culture studies comparing the expression of PD1 (**A)** and PD-L1 **(B)** on HIV+ CD8 T cells, stimulated with CEF (left) or Gag (right) peptides, in the absence (grey) or in the presence of autologous, HIV+ (black) or of HIV- (right) CD4+CD25^high^ T cells, (n = 8), (* p<0.05, Student’s T-test).
(TIF)

S5 Fig Treg inhibitory potential changes depending on HIVviral load. A representative experiment in which non-stimulated (a) or Gag-stimulated (b-d) HIV+ CD8 T cells from ART-naïve patient were cultured either in the absence of Treg, in the presence of autologous Treg, or in the presence of allogenic Treg from an ART+ patient with undetectable HIV VL (A) Individual data from co-culture and cross-culture studies comparing the inhibition of IFNγ expression by Gag-stimulated HIV+ CD8 T cells from ART-naïve patients in the presence of Treg from the same time point (left) or Treg from a different blood draw/or patient, after HIV VL suppression (right)(B).
(TIF)

S6 Fig PD1 and PD-L1 expression on CD8 T cells and Treg depending on HIV viral load. Individual data from co-culture and cross-culture studies comparing PD1 and PD-L1 expression by Gag-stimulated HIV+ CD8 T cells from ART-naïve patients in the presence of Treg from the same time point or Treg from a different blood draw/or patient, after HIV VL suppression (left panel). PD1 and PD-L1 expression by Treg from ART-naïve patients and Treg from a different time point/or patient, after HIV VL suppression (right panel).
(TIF)

S7 Fig The composition and inhibitory effect of Treg in HIV+ settings can be modulated. **A**. Inhibition of IFNγ expression by Gag-stimulated HIV+ CD8 T cells in the presence of autologous CD4+CD25^high^ T cells, set in co-culture *ex vivo* or after 18 hour preincubation of Treg with Gag peptides. Proportions of effector (CD25+FoxP3^high^CD45RA-) and naïve (FoxP3^low^CD45RA+) Treg before **(B)** and after (**C**) 18h preincubation with Gag peptides (a representative example of 4 separate experiments).
(TIF)

S8 Fig Flow cytometry analysis of PBMC before and after Treg depletion. PBMC before (upper panel) and after Treg-depletion with anti-CD25 Dynabeads as specified in Material and methods section (lower panel) were permeabilized and stained with a combination of FoxP3/CD25/CD127/CD4 mAbs to verify the efficiency of depletion. A representative example is presented; cells were gated on CD4 expression.
(TIF)
