# Supplementary figures and images for: Subset- and Antigen-Specific Effects of Treg on CD8+ T Cell Responses in Chronic HIV Infection
Source: PLoS Pathog. 2016 Nov 9;12(11):e1005995. doi: 10.1371/journal.ppat.1005995 (PMC5102588; doi:10.1371/journal.ppat.1005995)

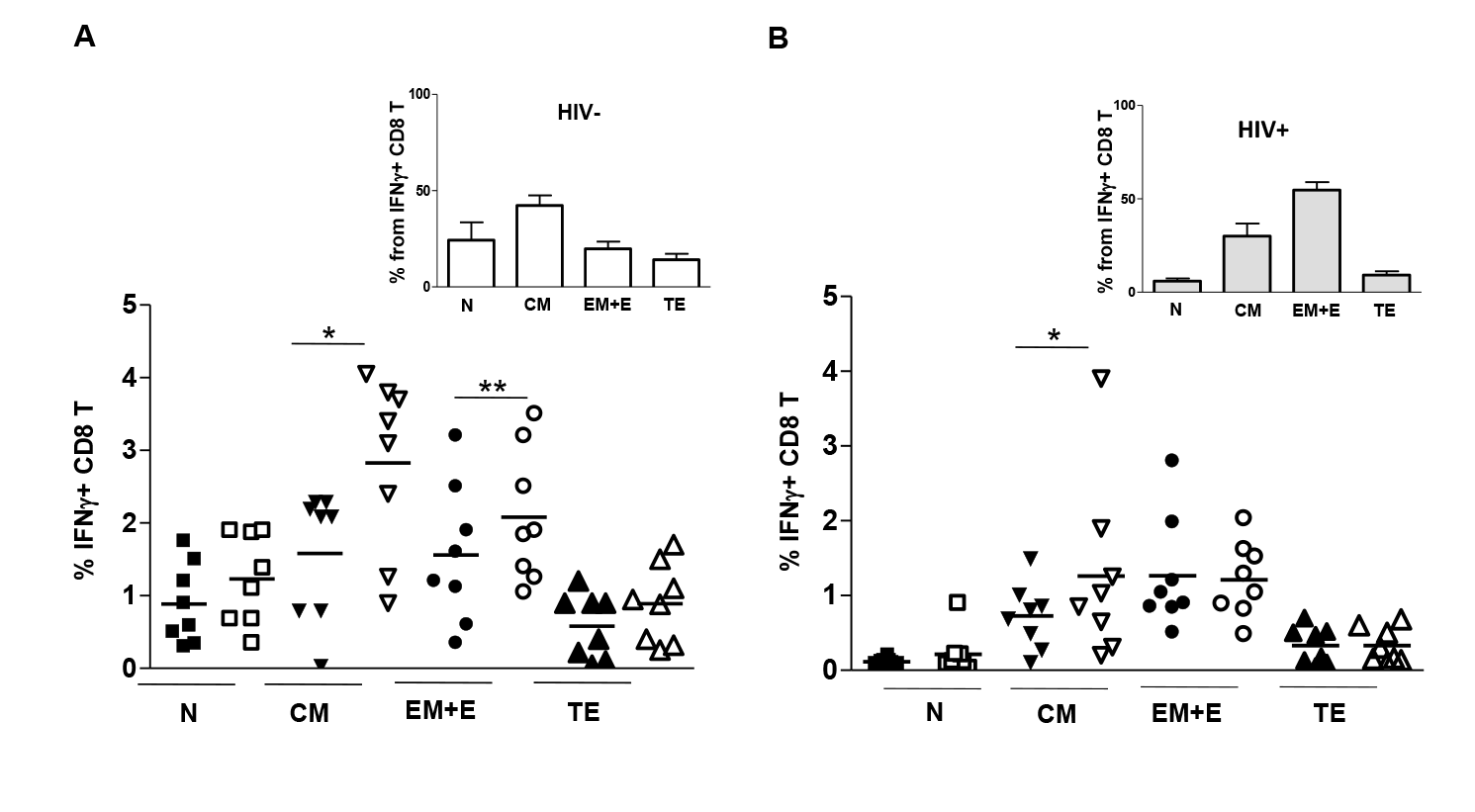

Supplement: S1 Fig — Individual data showing the effect of Treg on IFNγ secretion within CCR7/CD45RA-defined CD8 T subsets, after 18-hour stimulation with anti-CD3 antibodies in the presence (black) or in the absence (white) of Treg, in HIV- negative (A) and in HIV+ART-naïve (B) subjects. Embeded: pooled data showing the distribution of IFNγ+ CD8 T cells within the CD45RA/CCR7 defined subsets in HIV-(left) and in HIV+ settings (right). Bars represent mean ± SD. (* P <0.05, ** P<0.01, *** P<0.001, n = 10, paired Student’s T-test). (TIF) [file ppat.1005995.s001.TIF]

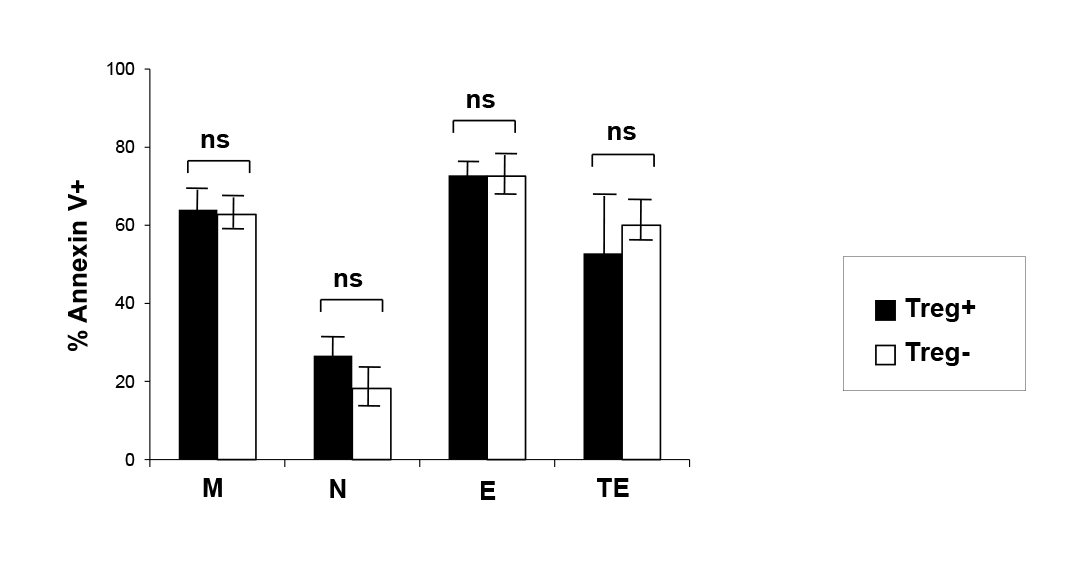

Supplement: S2 Fig — Pooled data about the percentage of AnnexinV+ cells within CD27/CD45RA-defined CD8 T subsets, after 48 hour stimulation with anti-CD3 antibodies in the presence (black) or in the absence (white) of Treg.(ns P >0.05, (n = 6, Student’s T-test). (TIF) [file ppat.1005995.s002.TIF]

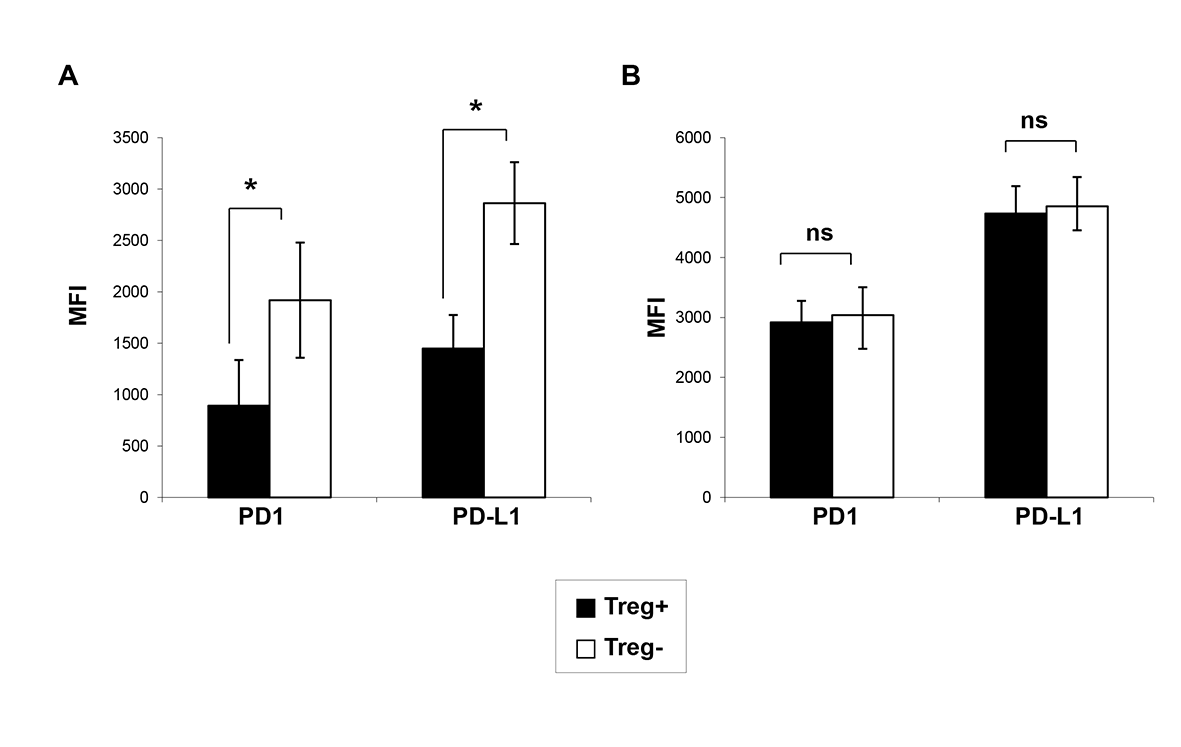

Supplement: S3 Fig — Pooled data on the levels of PD-1 and PD-L1 expression on CEF-specific (A) and Gag-specific (B) CD4 T cells stimulated overnight in the presence (black) or in the absence (white) of Treg. (* P <0.05,*** P <0.001, n = 10, paired Student t-test). (TIF) [file ppat.1005995.s003.TIF]

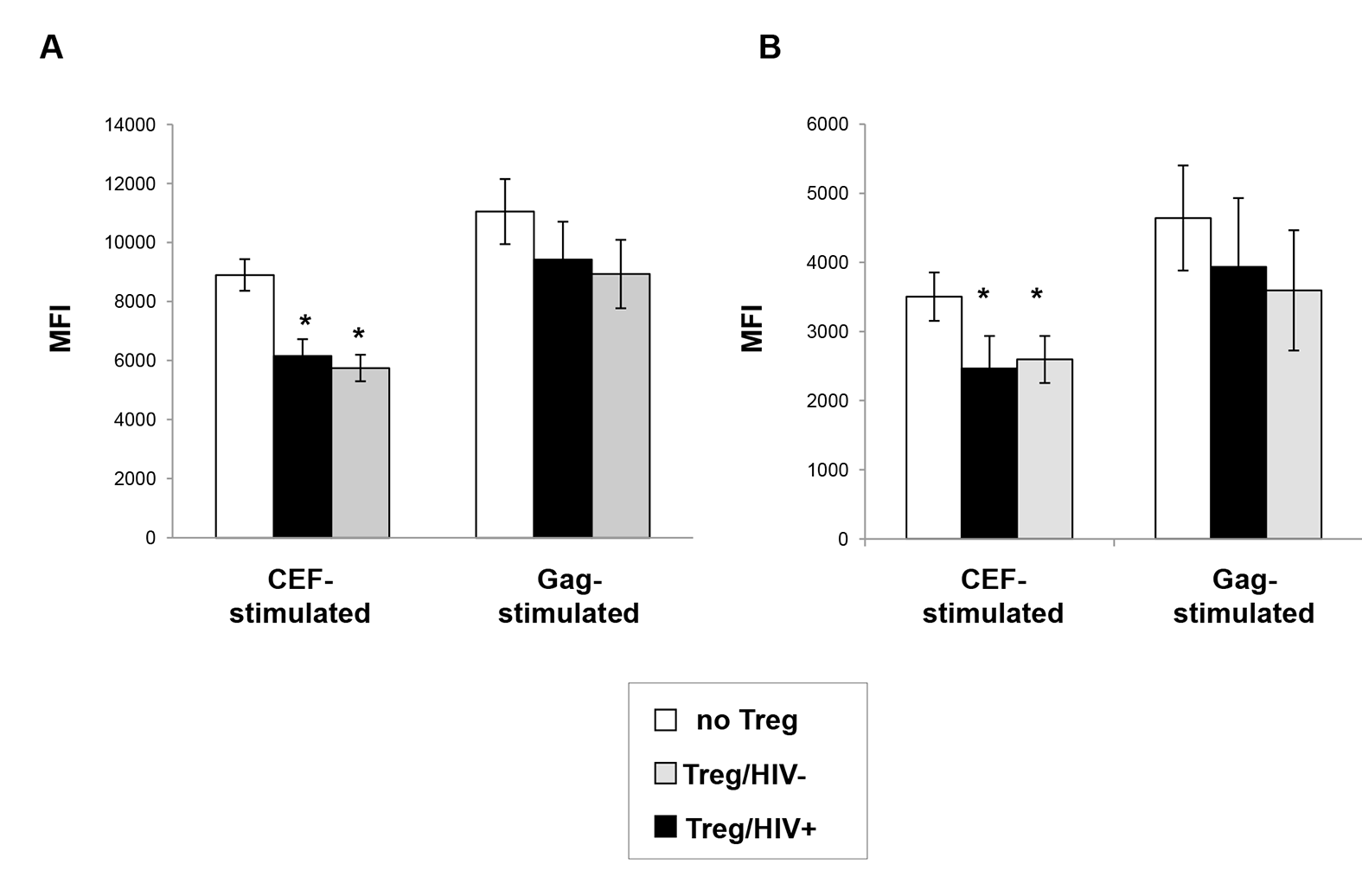

Supplement: S4 Fig — Individual data from co-culture and cross-culture studies comparing the expression of PD1 (A) and PD-L1 (B) on HIV+ CD8 T cells, stimulated with CEF (left) or Gag (right) peptides, in the absence (grey) or in the presence of autologous, HIV+ (black) or of HIV- (right) CD4+CD25high T cells, (n = 8), (* p<0.05, Student’s T-test). (TIF) [file ppat.1005995.s004.TIF]

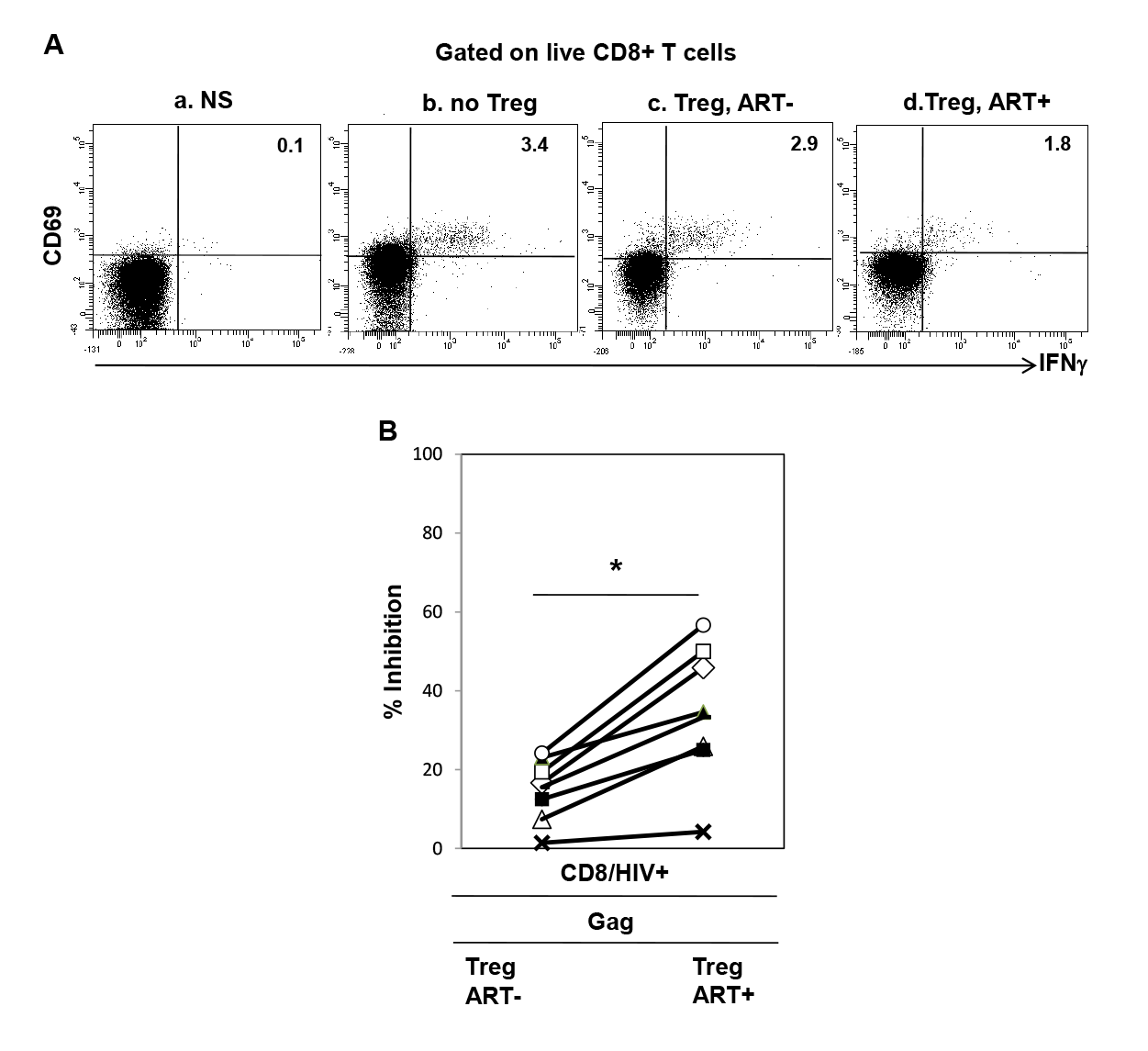

Supplement: S5 Fig — A representative experiment in which non-stimulated (a) or Gag-stimulated (b-d) HIV+ CD8 T cells from ART-naïve patient were cultured either in the absence of Treg, in the presence of autologous Treg, or in the presence of allogenic Treg from an ART+ patient with undetectable HIV VL (A) Individual data from co-culture and cross-culture studies comparing the inhibition of IFNγ expression by Gag-stimulated HIV+ CD8 T cells from ART-naïve patients in the presence of Treg from the same time point (left) or Treg from a different blood draw/or patient, after HIV VL suppression (right)(B). (TIF) [file ppat.1005995.s005.tif]

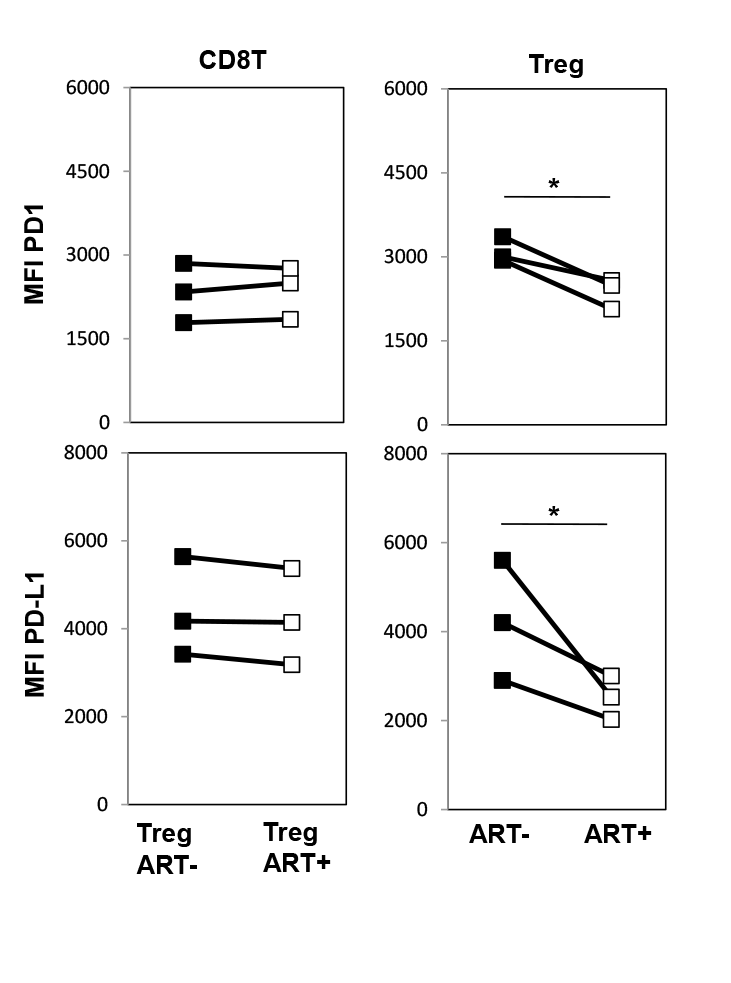

Supplement: S6 Fig — Individual data from co-culture and cross-culture studies comparing PD1 and PD-L1 expression by Gag-stimulated HIV+ CD8 T cells from ART-naïve patients in the presence of Treg from the same time point or Treg from a different blood draw/or patient, after HIV VL suppression (left panel). PD1 and PD-L1 expression by Treg from ART-naïve patients and Treg from a different time point/or patient, after HIV VL suppression (right panel). (TIF) [file ppat.1005995.s006.tif]

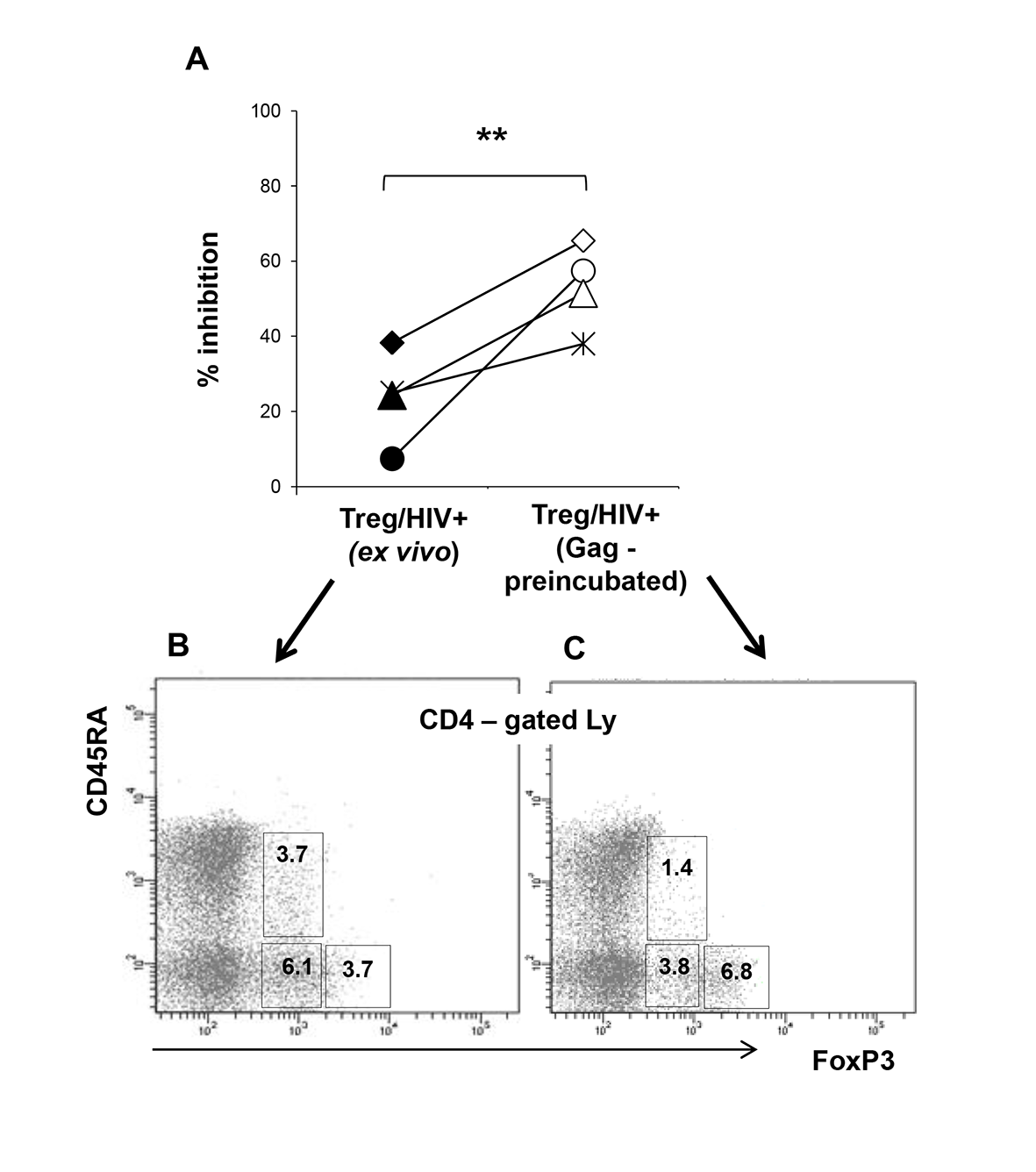

Supplement: S7 Fig — A. Inhibition of IFNγ expression by Gag-stimulated HIV+ CD8 T cells in the presence of autologous CD4+CD25high T cells, set in co-culture ex vivo or after 18 hour preincubation of Treg with Gag peptides. Proportions of effector (CD25+FoxP3highCD45RA-) and naïve (FoxP3lowCD45RA+) Treg before (B) and after (C) 18h preincubation with Gag peptides (a representative example of 4 separate experiments). (TIF) [file ppat.1005995.s007.TIF]

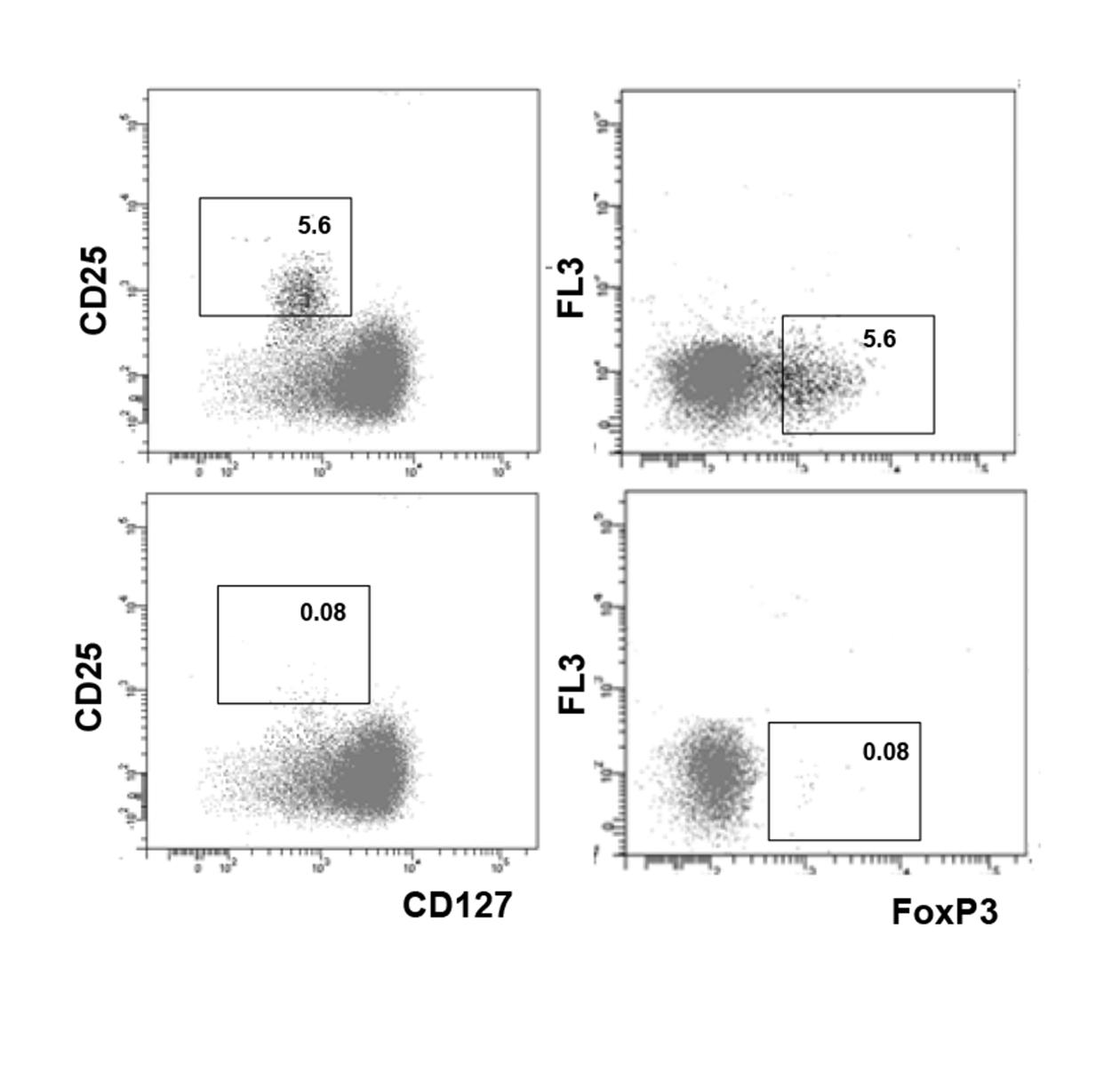

Supplement: S8 Fig — PBMC before (upper panel) and after Treg-depletion with anti-CD25 Dynabeads as specified in Material and methods section (lower panel) were permeabilized and stained with a combination of FoxP3/CD25/CD127/CD4 mAbs to verify the efficiency of depletion. A representative example is presented; cells were gated on CD4 expression. (TIF) [file ppat.1005995.s008.tif]
